# At the Intersection of Industry, Academia, and Government: How Do We Facilitate Productive Precision Livestock Farming in Practice?

**DOI:** 10.3390/ani9090635

**Published:** 2019-08-30

**Authors:** Brett C. Ramirez, Hongwei Xin, Patrick G. Halbur, Donald H. Beermann, Stephanie L. Hansen, Daniel C. L. Linhares, Joshua M. Peschel, Christopher J. Rademacher, James M. Reecy, Jason W. Ross, Timothy A. Shepherd, James E. Koltes

**Affiliations:** 1 Department of Agricultural and Biosystems Engineering, Iowa State University, Ames, IA 50011, USA; 2 Institute of Agriculture, The University of Tennessee, Knoxville, TN 37996, USA; 3 Department of Veterinary Diagnostic and Production Animal Medicine, Iowa State University, Ames, IA 50011, USA; 4 Department of Animal Science, Iowa State University, Ames, IA 50011, USA

**Keywords:** sensors, algorithms, implementation, economic feasibility, big data, rural and societal impacts, education and training

## Abstract

This commentary is a comprehensive synthesis of ideas generated from a workshop, hosted by Iowa State University, encompassing precision livestock farming (PLF) research and applications for industry–academia. The goal of this workshop was to demonstrate existing institution research and strategically propel further PLF development and industry adoption. Six key thematic areas were identified from participant discussion: sensors and algorithms, implementation, economic feasibility, data, rural and societal impacts, and education and training. These themes were used to focus discussion on identifying the new knowledge needed to drive implementation and examine current and future challenges of implementing PLF. At the convergence of industry and academia sits a unique opportunity to create mutually beneficial relationships that accomplish the individual needs of all parties. Productive PLF is currently hindered by numerous technical and non-technical challenges, but an increasing demand and optimistic outlook may result in rapid producer adoption. To foster harmonious partnerships among industry, academia, and government, a nexus at the intersection of multiple disciplines and basic/applied sciences is needed to thrust future success.

## 1. Introduction

Increased global demand for safe and secure animal-based protein has necessitated changes to livestock (including poultry) production systems and rapid development of new technologies to facilitate these unprecedented trends. Precision livestock farming (PLF) aims to use technology to continuously monitor the expression of an individual animal’s internal state (i.e., health, behavior, production/reproduction, neurophysiology, and physiology) and connect it with surrounding metadata (e.g., nutrition, environment, facility, management, genetics, etc.) to drive the discovery of solutions to deep scientific questions or pressing producer/societal needs. Technologies for PLF can be classified into real-time monitoring for control and on-farm decision making (e.g., robotic milking) or standalone systems to generate key data for basic science or widespread guidance across an industry (e.g., cameras to evaluate different farrowing stall configurations). Example PLF applications include addressing animal health (early disease detection), nutrition (optimal feeding or dietary impacts), welfare (resource allocation or handling), husbandry (animal management and care), breeding and phenomics (high-throughput phenotyping), environment (thermal/air quality), products (meat/egg/milk quality), etc. Such applications are truly boundless, and innovation is always thriving, but sector-wide adoption to address these grand challenges is rarely accomplished.

**Figure 1:**
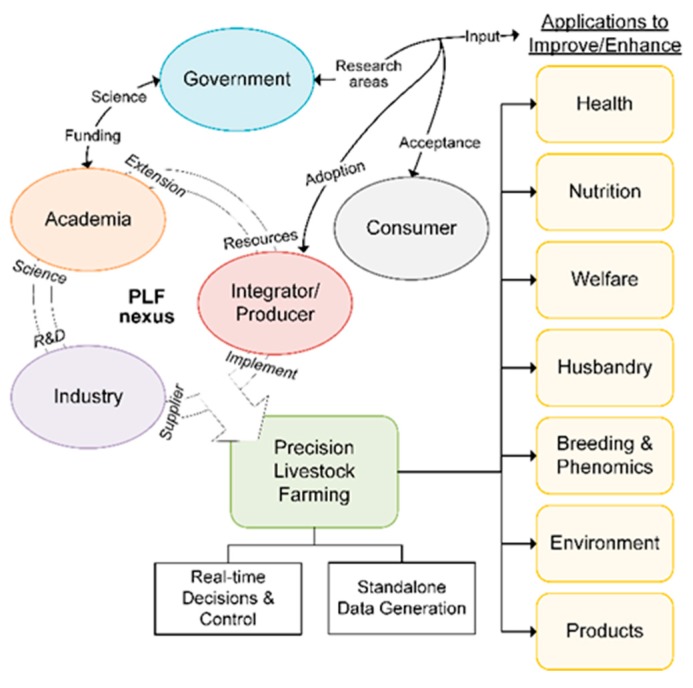
Key precision livestock farming (PLF) stakeholders and their interactions to drive and create applications for improving animal production systems. Government agencies shape the direction of PLF and have increasing invested in PLF areas. Social acceptance of PLF practices by consumers will also play a key role in research and development, as well as adoption.

Productive PLF requires stakeholders to jointly and independently overcome both technical and non-technical challenges. Education and training the future workforce in PLF requires an extensive network of educators and extension programs. Key PLF stakeholders identified are academia, producers, allied industries, government agencies, commodity groups, and consumers. The core development and implementation of PLF originates from allied industry, academia, and producers, while government agencies, commodity groups, and consumers can provide funding and shape direction. Industry will generally be defined as enterprises developing and marketing commercial technologies/equipment implemented by producers. Producers consist of those who oversee animal management, feeding, care, ownership, and marketing. Lastly, academia is public/private institutions focused on research, teaching, and extension. The specific goals of industry, academia, and producers are inherently different; however, there exists an intersection where all entities can prosper. Clear goals and intellectual exchange among entities are needed to span the transdisciplinary nature of PLF. A graphical depiction of this relationship and collaboration is illustrated in Figure 1.

To raise awareness and further foster public–private partnerships regarding the development and adoption of PLF technologies nationally and globally, a PLF workshop was hosted by the College of Agriculture and Life Sciences and College of Veterinary Medicine at Iowa State University (ISU) in December 2018. The workshop featured strategic speakers covering foundational research, funding opportunities from government agencies, and industry perspectives, as well as presentations from on-going research at ISU. A discussion concluded the workshop and participants responses to two strategic questions were used to catalyze this work. The overarching goals of this commentary are to formally disseminate this discussion, raise awareness among PLF stakeholders, and drive collaboration for adoption of productive PLF. Specific objectives of this commentary are to (1) highlight the unique industry, academic, and government perspectives on PLF development and adoption; (2) discuss where mutually beneficial collaborative relationships can be created between industry and academia; and (3) synthesize the future vision and challenges of implementing PLF.

## 2. Thematic Areas

The approximately 100 workshop participants (50 external and 50 internal to ISU) consisted of all levels of academia, producers, integrators, management services, and representatives from government agencies and commodity groups. Two strategic questions were prompted to facilitate discussion: (1) Based on what you have heard today, what new knowledge do we need to move forward with implementation of PLF in animal production? and (2) What challenges to implementation of PLF do you foresee? The responses to the questions were summarized and synthesized within six key thematic areas: sensors and algorithms, implementation, economic feasibility, data, rural and societal impacts, and education and training.

### 2.1. Sensors and Algorithms

A vital component of PLF is the ability of sensors and their accompanying algorithms to condition (i.e., convert to physical value) and interpret sensor signals. Sensors can detect the animal’s expressed internal state (i.e., physiological, behavioral, productivity, neurophysiological, and health) as well as measure or collect surrounding metadata (e.g., environment, facility, management, surroundings, etc.). Expression of the animal’s state may be sensed from on-animal (i.e., contact) sensors or non-contact sensors. Both categories have advantages and disadvantages that must be considered in the PLF scope. These sensors must be accurate, precise, cost-effective, reliable, and robust for livestock production system environments. These attributes are critical to providing high-quality inputs into the algorithms used to condition sensor signals and, ultimately, to interpreting/classifying the physical values to meaningful information from which assessment/decisions can be made. Sensor information must be processed in real-time with high accuracy, specificity, and sensitivity. Algorithm flexibility is critical to dynamic environments, facilities, and locations. Integrated, robust, and thoroughly tested algorithms are needed during lab/small-scale development to promote more effective integration at the commercial scale, and to promote the style of livestock production systems.

Today’s sensors and algorithms still face several challenges. Sensed information and measurements must be made into real-time, actionable advice/decisions, requiring complex technologies and analytical methods that are integrated with existing mechanical, human, and animal infrastructures. Most sensing technologies currently exist as standalone units; however, a multiplexing/integration of numerous PLF technologies (PLFTs) is needed to provide a unified structure of actionable/usable information for producers within the whole livestock production ecosystem. PLFTs measuring environment, nutrition, health, etc., will need to be integrated to create the general metadata. A diverse range of technology is needed to encompass the range of livestock production systems (e.g., group versus individual housing, size of operation, and species differences), and PLTs will need continuous innovation and development.

### 2.2. Economic Feasability

Developed PLFTs require techno-economic assessment to determine technical and economic performance of the product or process in which that product would augment. Example objectives include predicting the success of a product at different scales or performing an economic comparison of different products/technologies that provide a similar service. The value of a PLFT must be demonstrated at a stakeholder level to promote use and further development. Additional value in PLFT is needed beyond the data used to make day-to-day decisions. For example, producers would need to see an improvement in animal health (decrease in animal mortality and/or treatment rates) from actionable items generated by PLF.

Currently, the enhanced development of PLF is hindered by the limited availability of information to estimate return on investment and the value of information gained from some technologies. Another challenge regards its adoption in smaller operations when capital cost for new technology is often high, compared to larger operations that can purchase in large quantities to decrease costs. With new technologies, long-term serviceability can be a challenge as robustness of hardware is often unknown. A new skilled and trained workforce will be needed to install, calibrate, and manage PLF technology hardware for particular facilities as well as those to vertically manage and integrate data with other production data. Long-term costs associated with rapid changes in hardware are relatively unknown but must be factored into economic feasibility of implementation.

### 2.3. Implementation

At the forefront of implementing productive PLFTs is the need to fully understand adoption barriers for end users and the most economic methods to address them. This can strategically aim technology development towards having a greater likelihood of potential adoption. A match between animal operation scale and technology must be understood to ensure appropriate implementation. For example, small operations often have different production systems compared to large operations, which require PLFTs to be flexible.

Technologies must be thoroughly tested and proven to work under various environments and in a myriad of facilities. Implementation is often deterred by a lack of rigorous performance evaluation during initial development of PLFTs, leading to insufficient support from industry. Another major obstacle in implementation is producer acceptance. Producers frequently lack confidence in their ability to adopt PLF approach and use new technologies, as they often add additional management, maintenance, training, and time to existing operations. There is also a perception among producers that there is a possibility that technology will interfere with management priorities. As livestock production systems have over time been shifted toward large group management approaches, the mentality associated with focusing at the individual animal level may make it challenging for producers to assess value. The current and basic operations infrastructure could be an impediment, as most facilities are not designed to incorporate such PLF technologies. Further, the conversion of existing infrastructure could be cost-prohibitive when the upfront and operational costs of the PLFTs are included. Limited capability to link data from a variety of on-farm systems, equipment, records, etc., creates additional financial challenges to justify implementation.

### 2.4. Data

Data generated from PLFTs is valuable but requires cautious interpretation to be informative to all PLF stakeholders. Data integrity, integration, security, and usability are all critical aspects in the process of providing knowledge from PLF data. Extracting useful information from large quantities of these data will require careful, automated data parsing, standardization, database management, and data protection (i.e., maintenance of privacy and security) to provide robust predictions that are acceptable for industry. Database functionality and associated tools (e.g., software/API) for processing and management of large PLF data are needed for integrating different data types/streams from various sources. Given the heterogeneity of existing PLFTs, compatibility of different data tracking software is important, but only when the value of integrating data is greater than the cost of software integration. Development of actionable on-farm management software tools and research to generate predictive analytics to enhance production efficiency and animal health is also important. Recent advances in machine learning and artificial intelligence approaches will help quantify which PLF measurement features are most meaningful for management tools. Development of PLF management software will be impacted by the availability of open-source versus proprietary software for integration of multiple PLF data types. To facilitate the development of useful prediction and analytical tools, there is a need to grow the understanding and appreciation of the value of sharing PLF reference data from multiple operations in a common database. Access to such data references would allow for benchmarking and development of genetic selection and management tools, and engineering insights that could lead to animal industry-wide benefits.

There are several challenges associated with stakeholder and public perception of PLF and related data. Data can be viewed as a commodity, community resource, or its value can be misused/ manipulated for unintended uses. As such, defining data ownership and identifying appropriate means of protecting data is critically important. There needs to be development of security processes to protect producers and their data as well as protect the value of new technologies. Facilitating data sharing across producers, academia, and industry partners will be both a challenge and opportunity. Building trust among stakeholders will be critical to facilitate data sharing. Ideally, all stakeholders should come together to create agreements as to when and where data sharing would be acceptable. A reference database of shared data would allow for more powerful analyses in comparing benchmarks, allow for the development of more efficient algorithms, and produce greater community benefits in general. A shared data resource would also create some transparency for external stakeholders to better understand the intentions of PLF as a means of providing enhanced care over possible assumed objectives. Sharing data would also help reduce complexity by creating more standardization of how data streams are processed. Data standardization for both standalone and integrated technologies alike may need “data keys” that essentially act as data headers that can be passed between sources. Third-party data aggregators may be need to collected and standardized prior to being disseminating to invested parties. Additionally, uniform PLF terminology and methodologies adopted by organizations such as American Society of Agricultural and Biological Engineers (ASABE) or International Organization for Standardization (ISO) could aid in data standardization. Data shared from some technologies would come with more challenges than others, such as DNA sequence data from pathogen monitoring, large video data, etc.

### 2.5. Rural and Societal Impacts

Many PLFTs have incredible potential to enhance animal health and production efficiency, but there is also concern that they could be perceived in unintended ways by the public. Understanding the general public’s acceptance and concerns surrounding PLF will be important to facilitate acceptance and educate society on opportunities with these new technologies. Concerns regarding job loss, changes in animal welfare, and management are important to address. There is an opportunity in addressing these concerns to demonstrate the positive aspects of PLF by providing explanation of the social/public value of PLF in safe food production, prevention of antimicrobial resistance (AMR), and early diagnosis of sub clinically ill animals to enhance animal welfare and health. Development of PLF could also spur rural development by sustaining jobs and business in rural areas; however, from a technological standpoint, internet connectivity and capacity will need to improve in many places to achieve these objectives. All these improvements will require industry-academia partnerships, and the ability of academic researchers to work in these applied PLF applications. Incentives and support for early career researchers choosing to work in PLF will be important to ensure their willingness to perform research in these areas in the light of tenure and promotion demands. Universities need to provide support and rewards to researchers who can develop PLF researchers for application as well as insurances that these endeavors will be valued as part of their career development and promotion.

Challenges in implementing PLF also involve technological advancements and their public perception. Internet connectivity, particularly internet service provider infrastructure and costs, in rural areas as well as signal quality in and around structures are challenges. High-speed wireless (3G/4G/5G) is currently too costly for wide-scale implementation, and cellular density is often too low in rural areas for reliable data transfer. Faster low-power wide-area network deployment and ability to scale across rural areas is needed. Transparency in technology use will be important for public perception. For example, identification and tracking of animals may not be accepted if perceived as a means of watching people/employee behaviors versus the actual intent of monitoring animal health and behaviors. The ability to ensure employee rights and remove worker fears about privacy and trust represent potential barriers and will be important to address during PLFT implementation. Further, the public may see PLF as a means to increase the size of confinement operations and the number of animals within a facility. Thus, there is requisite need for education and discussion with the public to proactively explain how and why PLFT are used, the opportunities to enhance animal health and care, as well as the potential to create new technology driven jobs on farms and in rural settings. These growing challenges for PLF present opportunities to engage and educate the public more broadly on agriculture as well.

### 2.6. Education and Training

The current livestock production education model develops curriculum and training to meet specific demands, typically providing localized expertise relative to species (swine, beef, dairy, poultry, etc.), technical disciplines (management, nutrition, genetics, engineering, veterinary medicine, etc.), and regional production models (Northeast, Midwest, South, etc.). Meeting the current and future demands of developing, integrating, and managing PLFT will require a significant investment in the education and training of stakeholders at all levels. A robust PLF education program will require multi-disciplinary efforts that cut across traditional academic–industry boundaries to develop an infrastructure that can support the training of personnel who will (1) investigate and develop novel and applied PLFTs; (2) integrate PLF into production settings and manage hardware, infrastructure, and data requirements; and (3) manage the on-farm, animal–human–facility interactions and information generated with traditional and PLFT methods.

While the demand to integrate PLF is rapidly growing, the individual capacity of an academic unit, industry provider, or producer to provide education and training is significantly lagging. Challenges include expanding the inclusion of PLF fundamentals within established curricula; attracting non-traditional students with skills and expertise in data science, statistics, and technology applications into the field of livestock production; providing relevant and timely education to producers on the opportunities within PLF; and providing support for the training extension faculty and staff to develop PLF programming. To meet this challenge, an international effort leveraging academic, industry, and producer expertise is warranted to further develop methods to deliver critical education and workforce development programs.

## 3. Industry–Academia–Government Convergence

The specific goals of industry and academia are inherently different; however, there exists an intersection where both entities can prosper. Academia generally has expertise in the basic science and theory that can enable and lead to technology development. Due to the complex nature of animal production and technology, the expansive multi-disciplinary knowledge housed within academic institutions creates a ripe opportunity to solve the grand challenges faced by PLF. Desired outcomes for academia include scholarly dissemination of scientific results (often available to general public), and education program development (even though it is out of the scope and capacity of most academic institutions to create commercially scalable technologies). The latter desired outcome is dynamic, as incubators for start-up/spin-off companies and “research parks” are becoming more common.

Alternatively, industry stakeholders acknowledge the need to find pathways to scale-up the knowledge and technology developed by academia to commercially relevant products that can be deployed in their unique livestock production system. It is rare to find identical livestock production systems, in terms of company management, facilities, etc.; hence, PLF must work for their system and provide value to the entire production process. Outcomes for industry include lower production costs, improved animal welfare, reduced labor, as well as improved workforce knowledge and skills.

At the industry–academia intersection is the need for PLF commercialization companies to have the capability and capacity to understand the science generated by academia to customize and scale it to commercial livestock production systems. This PLF integrator (which can exist within industry or separately) works with both industry and academia. Mutually beneficial relationships need to be defined with clear outcomes and timelines. In addition, a skilled workforce trained in PLF development, implementation, and service provision, which can be educated by academia and acquired by industry, is a critical intersection.

Government support, especially at the global scale, plays a vital role in funding, facilitating, and disseminating PLF. High-risk financial investment is critical for PLF research and development by both academia and industry, and is generally achieved through government support. The degree of government support can vary considerably from country to country, but nonetheless, government programs can serve as the as the initial step in productive PLF and support achievement of PLF adoption.

### What Is the Monetary Value of Precision Technology Data?

**Figure 2:**
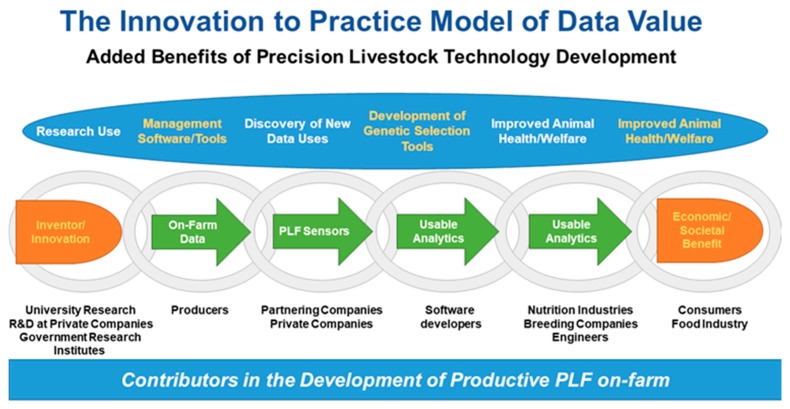
The innovation to practice model of data value.

Data analytics are revolutionizing agriculture, which begs the question, what is monetary value of data and who should reap the value? In the past, data have often been used for product development for industry or the common good for producers, but when and how should value be assigned? In Figure 2, we present an “innovation to practice model” as a means of conceptualizing the value of data. When industry uses data, should farmers/producers be compensated for this? Without producers, there would be no data. Likewise, without the technology, there would be no way to collect the data. The answer is not binary, as likely everyone in the data chain can claim ownership of some value. This is interesting when thinking about research, because companies sharing research has value, especially in the PLF space. Value should likely be ascribed to these data to provide industry with a monetary incentive, so that they can claim this value in their charitable/public donations. This also begs the following question: Should other entities pay to gain access to data? These are challenging questions that likely depend on the common good of the community. For example, in the livestock and poultry industry much of the genetic improvement that has been generated has come out of producers sharing data that benefits them through the creation of breeding tools, but industry also benefits because of the value of this shared data in marketing genetics. Both groups are invested to create products on farms and in the breeding industry, but perhaps in the arena of precision technologies with so many potential players, there is a need to define contributing entities and investors.

## 4. Future Vision and Challenges

What does the future hold regarding the needs and challenges in data handling and processing for productive PLF in both research and implementation? Infrastructure in the form of data processing pipelines and databases that work both on-farm and in the cloud will likely shape how PLF data is used. Systems are needed for rapid prediction to make real-time decisions as well as to achieve critical information for research and broader uses of genetic improvement, inform building designs, change management systems, etc. Data security will be a critical part of these systems. Development of data security and privacy software will have high value to facilitate data sharing and protection. The opportunity to create shared PLF data resources to fuel research will provide opportunities to develop new types of predictive analytics, to develop treatments for animals that will reduce antibiotic use (thus reducing AMR), and to enhance genetic improvement programs. Development of common data formats or easily accessed databases will be important in facilitating such advances. Importantly, issues of data ownership will need to be defined to allow for this kind of data sharing such that data is not trapped in proprietary software. If agreements can be developed to protect private businesses, innovative approaches will develop rapidly with varying data types, new methods, new technologies, and new approaches that may revolutionize PLF. In future research landscapes, precision technologies will also likely be used routinely for research to monitor animal welfare and phenomics.

Short-term as well as future challenges and opportunities of PLF largely revolve around network technology advances and communicating factual information to the public about how PLF will impact the future of agriculture. From a technology standpoint, setting up local networks and internet connections to capture data and transfer it for computation is critical. The ability to glean data for on-farm prediction models will reduce data transfer demands, but the research needed to identify such approaches will require large data transfer. Public concerns about how PLF will impact jobs, animal welfare, and farm sizes need to be addressed up front. PLF will likely create jobs in rural settings and new types of opportunities on farms. Fostering academic–industry–government–food supplier partnerships will be important in creating applied solutions with PLF. Building value at universities to support PLF researchers and interdisciplinary teams to tackle these challenges will be critical to spur the innovation needed to overcome challenges. The future development of PLF will depend on such interdisciplinary collaborations among animal scientists, veterinarians, molecular biologists, engineers, computer scientists, economists, sociologists, and industry representatives, to create applied solutions.

What does the future hold for PLF? As development costs continue to decrease, and training to handle the data expands, it is likely that PLF data will affect nearly every sector of animal production and lead to unique collaborations among animal scientists, engineers, computer scientists, and veterinarians. This convergence of different disciplines will be essential to solve complex problems in animal production systems. Among many other potential applications, an example is promoting animal health with antibiotic stewardship via real-time monitoring for early onset of disease outbreak detection. Precision animal nutrition can be achieved with the capture of real-time water and feed intake, body weight estimation and growth prediction, and precision feeding systems. High standards of animal welfare could be achieved via autonomous classification of abnormal behaviors and early lameness detection and prevention. General husbandry and animal management could be substantially augmented and improved through in-depth study of resource allocation (e.g., feeder space, floor space, perch usage, number of drinkers/feeders, etc.), digital tools to augment caretaker knowledge for performing daily chores (identifying sick pigs, performing euthanasia), and early warning advice for equipment failure. All new phenotypes and environmental measurements collected by precision technologies could be combined with existing metadata to result in improved genetic selection. Environmental stewardship and air quality may be enhanced with real-time gas and particulate monitoring for quantifying emissions, demand-based thermal environment and air quality control for optimal production, and water quality and manure-nutrient content monitoring. Food traceability and the safety of animal products from farm to fork could reach new levels of transparently and accountability. Lastly, accomplishment of these examples requires skilled people in many disciplines, backgrounds, and sectors; hence, the educational infrastructure must exist to train these people through a PLF certification programs for professions in industry and transdisciplinary undergraduate and a graduation PLF curriculum.

As researchers become more multidisciplinary and develop more innovative ideas, the technology and data generated from PLF will be at the forefront of solving grand challenges in animal agriculture for many years to come.

